# A Dual Reporter Mouse Model of the Human β-Globin Locus: Applications and Limitations

**DOI:** 10.1371/journal.pone.0051272

**Published:** 2012-12-14

**Authors:** Petros Papadopoulos, Laura Gutiérrez, Reinier van der Linden, John Kong-A-San, Alex Maas, Dubravka Drabek, George P. Patrinos, Sjaak Philipsen, Frank Grosveld

**Affiliations:** 1 Department of Cell Biology, Erasmus MC, Rotterdam, The Netherlands; 2 Department of Pharmacy, University of Patras, Patras, Greece; Università degli Studi di Milano, Italy

## Abstract

The human β-globin locus contains the β-like globin genes (*i.e.* fetal γ-globin and adult β-globin), which heterotetramerize with α-globin subunits to form fetal or adult hemoglobin. Thalassemia is one of the commonest inherited disorders in the world, which results in quantitative defects of the globins, based on a number of genome variations found in the globin gene clusters. Hereditary persistence of fetal hemoglobin (HPFH) also caused by similar types of genomic alterations can compensate for the loss of adult hemoglobin. Understanding the regulation of the human γ-globin gene expression is a challenge for the treatment of thalassemia. A mouse model that facilitates high-throughput assays would simplify such studies. We have generated a transgenic dual reporter mouse model by tagging the γ- and β-globin genes with GFP and DsRed fluorescent proteins respectively in the endogenous human β-globin locus. Erythroid cell lines derived from this mouse model were tested for their capacity to reactivate the γ-globin gene. Here, we discuss the applications and limitations of this fluorescent reporter model to study the genetic basis of red blood cell disorders and the potential use of such model systems in high-throughput screens for hemoglobinopathies therapeutics.

## Introduction

The human β-globin locus spans ∼70 Kb containing the regulatory sequences of the Locus Control Region (LCR) and the β-like globin genes situated in the same order as they are expressed throughout ontogeny [5′- *HBE (ε)* - *HBG2 (Gγ)* - *HBG1 (Aγ)* - *HBD (δ)* - *HBB (β)* -3′]. Mice carrying a “minilocus” transgene, containing the essential distal regulatory elements surrounding the *HBB* gene, express it at levels equivalent to the endogenous mouse β-globin and have given valuable information related to regulatory regions, position-independent and copy number-dependent expression [1]. Mice bearing the entire human β-globin locus have been a useful model to understand developmental expression patterns of the five functional human β-like globin genes [2]. Combined studies on human and mouse globins have revealed common and different aspects of human and mouse erythropoiesis. While in human there are two globin switches (*HBE* to *HBG1/HBG2*, occurring in the transition from primitive to definitive erythropoiesis, and *HBG1/HBG2* to *HBB*, occurring in definitive erythropoiesis around the time of birth), there is only one switch in mouse occurring at the time of transition between primitive and definitive erythropoiesis [3]. Expression of the murine βminor and βmajor genes starts in the primitive cells and peaks at definitive stage when the embryonic ε and βh1 are no longer expressed [4]. Early studies on the human β-globin locus transgenic mice demonstrated expression of γ-globin in the embryonic stage as well as in the early fetal liver of the mouse whereas the β-globin gene was expressed in the fetal and adult stages [2].

The transition from γ- to β-globin during development involves both genes in a competitive model of transcriptional regulation [5]–[7]. A number of studies have confirmed the complexity of this process and have pointed out the need of a flexible and reliable model that facilitates high-throughput analyses. This has been attempted by substituting the globin genes with fluorescent proteins under the regulatory elements of the β-globin locus [8], [9]. However, due to the length of the complete human β-globin locus, most of the reporter constructs used to date are partial representations of the locus that can be easily manipulated and introduced in erythroid cells by standard transfection techniques. Missing genomic sequences may affect expression of the globin genes, which may lead to unreliable results in terms of physiological relevance. Therefore, such reporter assays should ideally be performed in the context of the complete locus [10], [11]. The generation of a reporter mouse model, where erythropoiesis can be studied at the developmental level, under steady-state and under stress conditions, would therefore be highly advantageous, in particular since erythroid cell lines generated from these mice could be used for high-throughput screening purposes.

Here we describe the generation of two lines of transgenic mice carrying the full-length β-globin locus with dual fluorescent reporter genes, HBG1-GFP|HBB-DsRed (γGFP/βDsRed) and HBG1-GPA-GFP|HBB-DsRed (γGPA-GFP/βDsRed). These lines differ in the cellular localization of the GFP reporter signal for γ-globin, *i.e.* cytoplasmic or at the membrane surface respectively. These mice allow *in vivo* tracing of *HBG1* (γ-globin) gene expression during development by flow cytometry or fluorescent microscopy. They can be used to test potential treatment modalities aimed at reactivating the expression of γ-globin in the adult stage. Additionally, we have generated fetal liver cell lines derived from these transgenic mice for *in vitro* experiments, especially for functional screens with libraries of chemical compounds, antibodies and shRNA clones, and molecular studies that require large amounts of cells. In addition, the limitations of this and other current reporter mouse models will be discussed with the aim to shed light to the generation of future globin reporter systems.

## Materials and Methods

### Ethics Statement

Animal housing, mouse strains (C57BL/6, FVB), knockout mice (p53) and transgenesis procedures used for the purposes of this study fall within the norms set by the ethics committee of Erasmus Medical Center (Rotterdam, The Netherlands). The *in vivo* experiment performed in this study and previously described by Rupon et al [12] which includes intraperitoneal administration of phenylhydrazine (PHZ) and azacytidine (AZA) was permitted under the protocol (DEC Nr. EMC2103, 138-10-08).

The ethics committee of Erasmus Medical Center (Rotterdam, The Netherlands) has approved all experimental protocols used to complete this study.

### Modification of the human β-globin locus in a PAC vector and generation of transgenic mice

The *HBG1* and *HBB* genes (PAC2 vector) were modified at the first *ATG* of the transcript by introducing the EGFP-N2 (720 bp, Clontech) or GPA-(EGFP-N2) and DsRed2 (700 bp, Clontech) cDNA respectively followed by a stop codon. Mouse Glycophorin A cDNA (GPA, 507 bp) was cloned and modified by introducing the EGFP-N2 cDNA 114 bp downstream from the start site of transcription by mutating a single base (Stratagene QuikChange II Site-Directed Mutagenesis Kit, Agilent Technologies) thus creating a *BamHI* site. The modified globin genes were used to subsequently replace the endogenous genes in the PAC2 vector by homologous recombination [13].

Fertilized oocytes from C57BL/6 mice were injected with *SceI* linearized modified β-globin locus devoid of vector sequences and three transgenic lines were generated, two of them from the EGFP-N2 and one from the GPA-(EGFP-N2) construct.

### Southern Blotting, S1 nuclease protection assays and qPCR

Southern blotting was performed for mapping the modified β-globin locus after each recombination step to ensure integrity of the construct. DNA was digested with different restriction enzymes and run on 0.6% agarose gel. The membrane was hybridized at 65°C with the two cosmid probes, cosLCR-ε and cosγγδβ [2] spanning the human β-globin locus.

S1 RNA analysis of murine globin expression was performed as described previously [2].

RNA from fetal liver cells was isolated using Trizma reagent (Sigma). cDNA was synthesized with SuperScript-II kit (SS-II, Invitrogen). Real Time quantitative PCR analysis was performed on an iCycler (BioRad) or Opticon I (MJ Research) system monitor using SYBR Green I. Enrichment for a specific sequence was calculated as 2 to the power of (−ΔCT), using Hypoxanthine-guanine phosphoribosyltransferase (Hprt1) and Glyceraldehyde-3-phosphate dehydrogenase (Gapdh) as housekeeping reference transcripts.

Primers used:

GFP: 5′-gacgacggcaactacaagac-3′ and 5′-cgttgtggctgttgtagttgt-3′.

DsRed: 5′-aagggcgagacccacaag-3′ and 5′-gtgtagtcctcgttgtggga-3′.

Hprt1: 5′-agcctaagatgagcgcaagt-3′ and 5′-atggccacaggactagaaca-3′.

Gapdh: 5′-cctgccaagtatgatgacat-3′ and 5′-gtcctcagtgtagcccaag-3′.

### Flow cytometry analysis

Analysis of embryonic or adult blood and primary fetal liver cells or cell lines was performed with either a FACSAria or a FACScan (Becton Dickinson, BD). Cells were resuspended in 1% BSA/PBS. Hoechst or 7-AAD (Molecular Probes) were used to stain dead cells. Antibodies for CD71-PECy7 and CD117-APCCy7 were purchased from BD Biosciences. Analysis of the data obtained was performed with FlowJo software (Tree Star Inc).

### Culture of primary fetal liver cells and generation of transgenic cell lines

Fetal liver cells were cultured in Stem Pro media (Invitrogen) in the presence of erythropoietin (Epo, 1 U/ml), stem cell factor (SCF, 100 ng/ml) and dexamethasone (Dex, 10^−6^ M; Sigma). All transgenic mice were bred with p53 knockout mice [14] to homozygosity to generate fetal liver cell lines as described [15], [16]. Differentiation assays were performed in the presence of Epo (10 U/µl), human transferrin (50 mg/ml, SciPac) and cell size was monitored with a CASY cell Counter (Roche). Within 48 h the cells homogeneously reduced their size and started producing hemoglobin. Globin expression of transgenic mice and cell lines was followed by flow cytometry, fluorescence microscopy IX-70 (Zeiss) or confocal microscopy (Zeiss) and standard RNA analysis techniques.

Hanging drop cultures were performed as described [17].

100 mM Hydroxyurea (HU, Sigma), 300 nM 5-Azacytidine (AZA, Sigma) and 200 mM RB7 (SPECS, Delft, The Netherlands) were added when indicated.

### Azacytidine treatment of transgenic mice

Transgenic and wild type C57BL/6 mice were treated as described [12]. Blood was resuspended in PBS and analyzed with a FACSAriaII. Bone marrow was cultured in hanging drop cultures [17] and analyzed by flow cytometry as mentioned before.

### Viral transduction

Knockdown experiments for different target genes were performed in transgenic fetal liver cell lines and in primary cells with concentrated virus. The shRNA plasmids are part of the TRC1 Mission shRNA library (Sigma). Lenti viruses were produced using a third generation system [18]. 20 µg of DNA plasmid from the TRC1 library together with helper plasmids were co-transfected in 293T cells with Polyethylenimine (PEI, Sigma) and supernatant was harvested during the next three consecutive days followed by filtration and centrifugation at 20 K rpm for 2 hours at 4°C.

Fetal liver cells were transduced with virus and grown under puromycin (1 µg/ml) selection for a maximum of one week.

### Western blots

Nuclear or whole cell protein extracts were semi-dry blotted to PVDF (Millipore) membrane and staining performed according to standard protocols. The ECL kit (Amersham Biosciences) was used to develop the membrane.

Primary antibodies used were: cMyb (SantaCruz-516), Bcl11a (Santa Cruz-56013) Hdac3 (AbCam-32369), Fop (KT64; Absea Biotechnology), Actin (sc-1616; Santa Cruz Biotechnology) and Nucleophosmin (Abcam-10530) all in 1∶2000 dilution. Anti-mouse IgG coupled to HRP (1∶20000) was used for detection.

### Statistical analysis

2-tailed T-test was performed when indicated.

## Results

### Modification of the human β-globin locus and generation of transgenic mouse lines carrying the dual reporter

**Figure 1:**
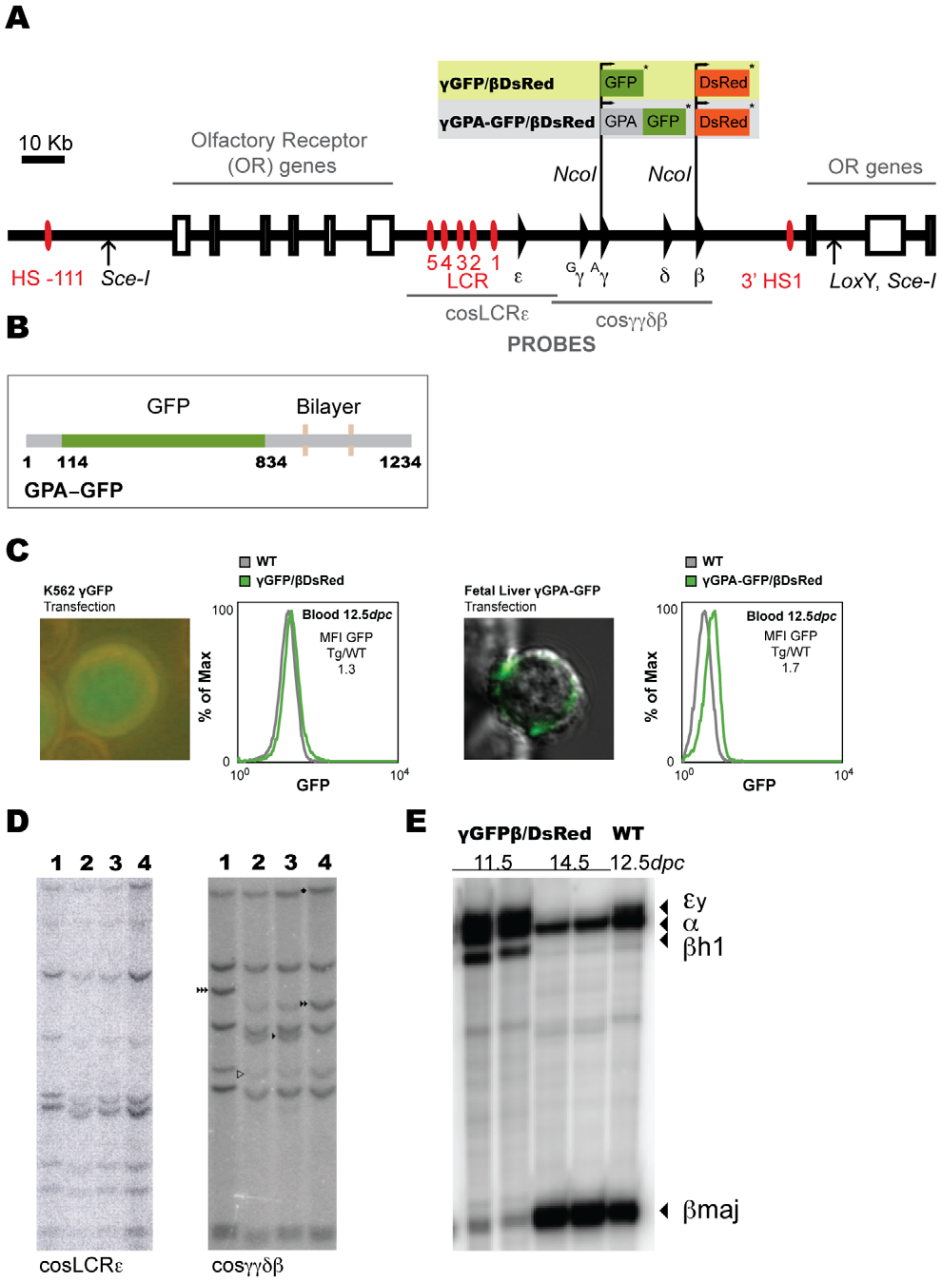
Modification of the human β-globin locus and generation of transgenic dual reporter mouse lines. (**A**) The human β-globin locus (*SceI* flanked) PAC used for the modifications made in the γ- and β-globin genes. GFP and DsRed were introduced in the ATG (+1) position of the transcripts followed by a stop codon (*). (**B**) Schematic representation of the GPA-GFP construct. The grey numbered stretches of the cartoon (1–114, 834–1234 bp) represent the glycophorin-A cDNA and the green stretch represents the GFP cDNA (114–834). The bilayer represents the transmembrane part of the protein, thus the GFP is expressed in the extracellular part of the fusion GPA-GFP protein. (**C**) Representative picture of K562 cells transfected with the γGFP/βDsRed modified human β-globin locus to check expression of γ-globin and flow cytometry analysis of GFP expression in 12.5*dpc* embryonic blood of γGFP/βDsRed transgenic embryos (left). Representative picture of fetal liver cells transduced with the γGPA-GFP construct to check expression of GFP protein in the plasma membrane and flow cytometry analysis of GFP expression in 12.5*dpc* embryonic blood of γGPA-GFP/βDsRed transgenic embryos (right). Mean fluorescence intensity (MFI) ratio is indicated in both graphs. (**D**) Southern blot of both mouse transgenic lines (γGFP/βDsRed and γGPA-GFP/βDsRed). Tail genomic DNA was digested with *SacI* restriction enzyme and hybridized with cosLCRε (left) and cosγγδβ (right), as previously described [2]. Lane 1: γGPA-GFP/βDsRed tail DNA, Lanes 2, 3: mouse line PAC8 carrying the human β-globin locus and Lane 4: γGFP/βDsRed tail DNA. Symbol ▹ indicates end fragments, ▸ HGG1 3.6 Kb *SacI* fragment, ▸▸ HGG1-GFP 4.3 Kb *SacI* fragment, ▸▸▸ HGG1-GPA-GFP 4.9 Kb *SacI* fragment, ⧫ β-DsRed modification (16.4 to 17 Kb fragment). (**E**) S1 nuclease protection analysis of mouse globin expression of WT and transgenic mice at different developmental stages as indicated.

In order to generate a mouse model where the expression of human globin genes can be followed by fluorescence, we introduced the GFP cDNA (EGFP-N1, Clontech) in the first ATG codon of the *HBG1* gene (PAC2 vector) and the DsRed cDNA (DsRed2, Clontech) in the first ATG codon of the *HBB* gene (PAC2 vector), both ending by a stop codon (Figure 1A) without replacing the endogenous transcripts. The same strategy was followed to generate the second transgenic line, which has the GFP fused to the erythroid-specific membrane protein Glycophorin A (GPA) as the γ-globin reporter (Figure 1A and B). GPA was chosen in order to express the GFP protein in the erythroid cell membrane rather than in the cytoplasm, to reduce possible fluorescence quenching due to high hemoglobin concentrations in the cytoplasm (Figure 1C). Since GPA-GFP has to be efficiently transported to the plasma membrane we tested the functionality of the fusion protein GPA-GFP by transducing a fetal liver cell line with a GPA-GFP lentivirus. Expression of GFP in the plasma membrane was confirmed by confocal microscopy (Figure 1C).

The modified human β-globin locus devoid of any sequences of the PAC2 vector (*SceI* digest, Figure 1A) was injected in fertilized C57BL/6 oocytes to generate transgenic mice. Extensive mapping of the modified locus was performed after each modification in bacteria and on tail DNA of founders by Southern blotting (data not shown and Figure 1D) using as probes two cosmids spanning the β-globin locus (∼70 Kb), cosLCRε and cosγγδβ, as described [2]. Two transgenic lines γGFP/βDsRed were obtained with two and one copy of the transgene respectively. Three founders of the γGPA-GFP/βDsRed construct were obtained, one of which transmitted the transgene to the next generation and was found to carry two copies of the transgene. Mouse globin gene expression during development was examined in transgenic embryos of both lines carrying two copies by S1 nuclease protection assays, and was found to be normal in terms of globin gene expression at the respective developmental stage (data not shown and Figure 1E).

As revealed by flow cytometry analysis (Figure 1C and data not shown), the difference between the two constructs (γGFP/βDsRed and γGPA-GFP/βDsRed, both carrying two copies) was small with respect to GFP detection in 12.5*dpc* blood cells. However, expressing the GFP protein on the cell surface, *i.e.* away from the cytoplasmic hemoglobin-rich environment of the erythrocyte, facilitates the detection of fluorescence signals on erythroid cells upon differentiation. For this reason, we chose to perform the developmental analysis of reporter expression with the γGPA-GFP/βDsRed transgenic mice.

### Human HBG1/HBB-globin gene reporter expression in γGPA-GFP/βDsRed transgenic mice during development

**Figure 2:**
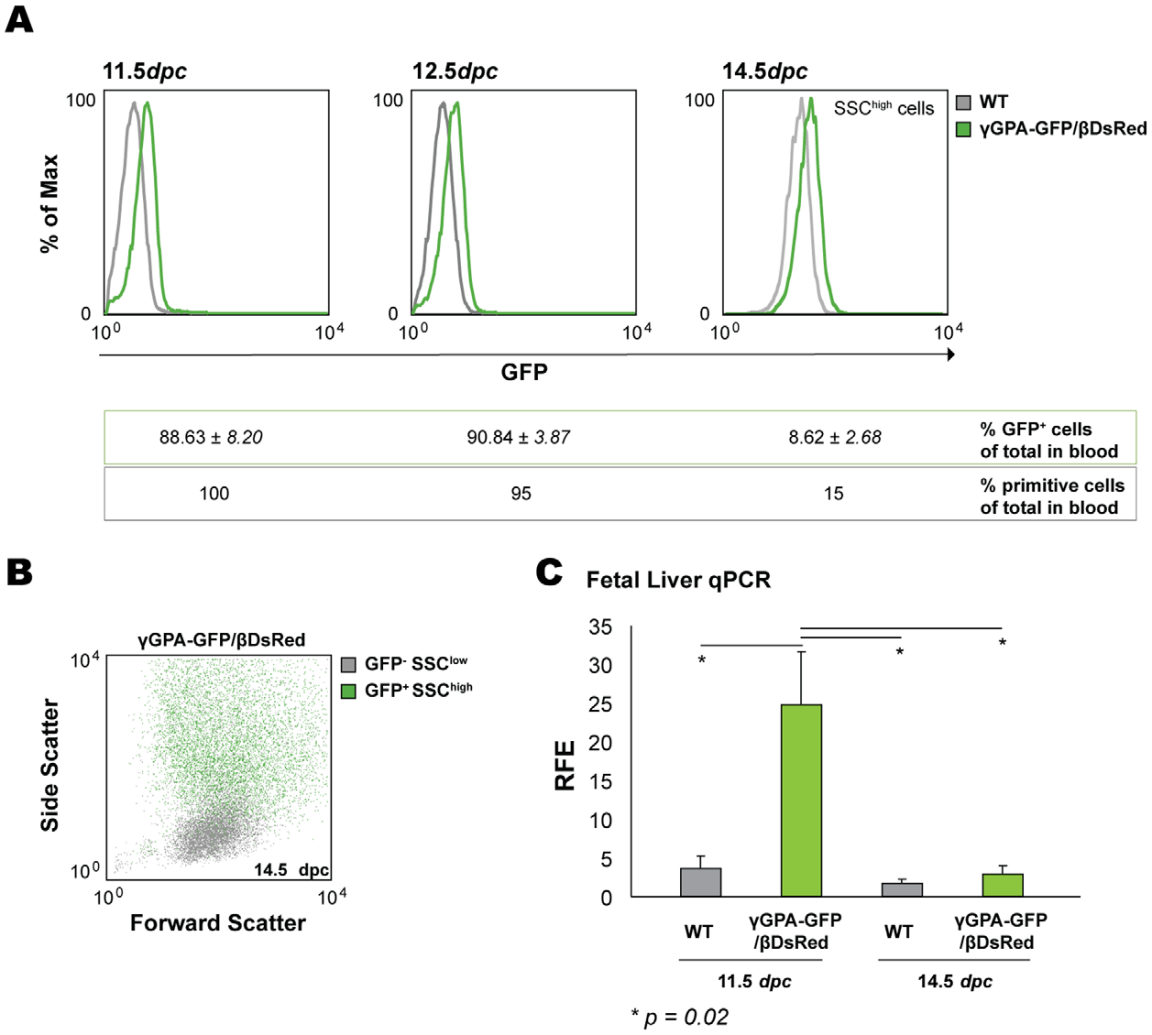
Analysis of GFP (γ-globin) expression during development. (**A**) Histogram overlay of embryonic blood from transgenic and WT embryos in the GFP axis. The percentages of positive GFP cells and primitive cells for each developmental stage are included in the table underneath (SSC: side scatter). Differences in the MFI of WT cells amongst the three histograms shown are due to the developmental stage and the gated cells plotted (i.e. SSC^high^ in the third histogram). (**B**) Dot plot depicting embryonic blood of representative γGPA-GFP/βDsRed transgenic mice at 14.5*dpc* in which the GFP^+^ population is depicted as green dots against the Forward (FSC) and Side Scatter (SSC). GFP^+^ cells (8.62±2.68%) are SSC^high^, *i.e.* primitive cells. (**C**) qPCR analysis of GFP expression in WT and transgenic fetal liver cells at 11.5*dpc* and 14.5*dpc*. RFE is relative fold enrichment. Average and standard deviation derived from three mice per group is depicted. T-test was performed to calculate the *p* values.

The flow cytometry analysis that was carried out in embryonic blood and fetal liver from embryos at different developmental stages was in concordance with previous studies of human globin gene expression in the mouse [2]. We could detect GFP expression in practically all circulating erythroid cells at 11.5*dpc* and 12.5*dpc*, after which the percentage of GFP-positive cells started to decline (Figure 2A and B). At 14.5*dpc* we could still detect GFP expression in a subpopulation of cells although their percentage was low (8.62%). These cells were side scatter high (SSC^high^) indicating that they were primitive cells still in the circulation (Figure 2A and B) [9]. Furthermore, qPCR analysis of GFP transcript in WT and γGPA-GFP/βDsRed fetal liver cells at 11.5*dpc* and 14.5*dpc* revealed GFP transcripts present in transgenic fetal liver cells at 11.5*dpc* (derived from primitive circulating cells), but not at 14.5*dpc* (Figure 2C).

**Figure 3:**
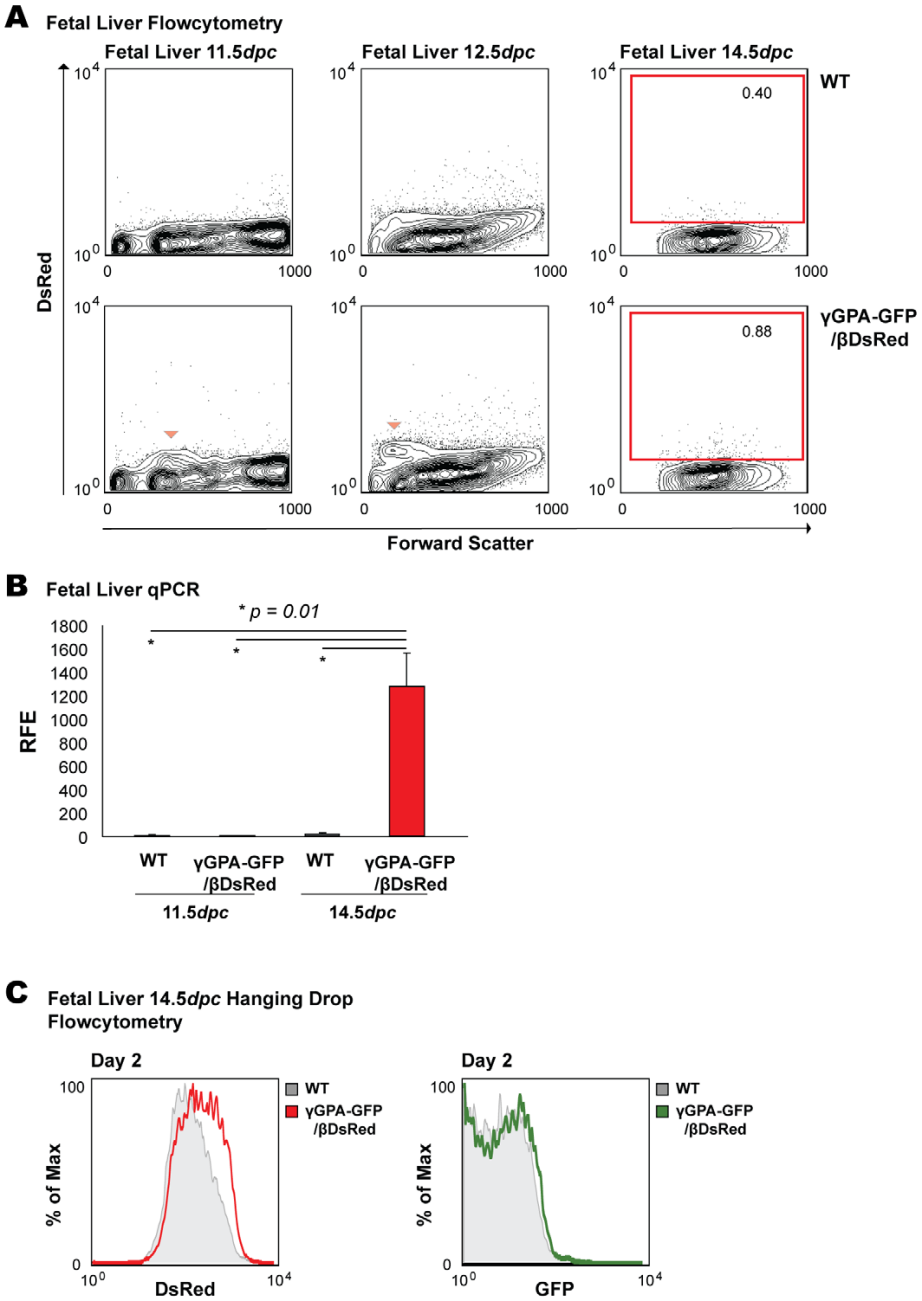
Analysis of DsRed (β-globin) expression during development. (**A**) Flow cytometry analysis of fetal liver of γGPA-GFP/βDsRed transgenic mice during development. Arrowhead at 11.5*dpc* and 12.5*dpc* indicates the DsRed positive population. Representative data are depicted. (**B**) qPCR analysis of DsRed expression in WT and transgenic fetal liver cells at 11.5*dpc* and 14.5*dpc*. RFE is relative fold enrichment. Average and standard deviation derived from three mice per group is depicted. T-test was performed to calculate the *p* values. (**C**) Histogram overlays of DsRed and GFP expression in 14.5*dpc* fetal liver cells cultured for 2 days in hanging drops. DsRed expression is detected in transgenic cells differentiated *in vitro* when compared to WT while GFP is not. Representative data are depicted.

A small fraction of fetal liver cells (Figure 3A, arrowhead) is positive for DsRed at 11.5*dpc* and 12.5*dpc*. At 14.5*dpc*, when expression of β-globin gene is high, it is extremely difficult to detect DsRed positive cells. When analyzing fetal blood for the expression of DsRed, we found no expression at 11.5*dpc* and 12.5*dpc*, as expected; however, as in the fetal liver, we could not detect DsRed in 14.5*dpc* blood (data not shown). Since the absorbance spectrum of hemoglobin [19]–[21] overlaps with the emission spectrum of DsRed (550 nm–700 nm), it seems likely that fully hemoglobinized cells would quench (cytoplasmic) DsRed emission in blood definitive cells and in the fetal liver context [22]. Indeed, qPCR analysis of DsRed transcript in WT and γGPA-GFP/βDsRed fetal liver cells in mice revealed DsRed transcripts present at 14.5*dpc*, but not at 11.5*dpc* (Figure 3B). Due to the fact that 14.5*dpc* fetal livers contain a relatively high proportion of mature, fully hemoglobinized erythroid cells, and in order to see whether we could detect DsRed expression in a more synchronous differentiating population outside the fetal liver context, we differentiated fetal liver cells *in vitro* in a hanging drop culture system [17]. As expected, we were able to detect DsRed expression, but not GFP expression, after two days of differentiation of 14.5*dpc* fetal liver cells in hanging drop cultures (i.e. β-globin is being expressed) (Figure 3C). Therefore, the combination of dual reporter fetal liver cells and the hanging drop culture system provides a tool for screening chemical, antibody or shRNA libraries in a low-cost manner.

### *In vivo* treatment of transgenic mice with 5-Azacytidine

Rupon and coworkers have described that stress erythropoiesis induction in combination with 5-azacytidine treatment in β-globin YAC mice resulted in reactivation of γ-globin, as measured by mRNA expression in circulating reticulocytes [12]. In order to assess whether we could detect those γ-globin expressing cells directly in the adult blood by flow cytometry, γGPA-GFP/βDsRed as well as WT mice were treated with phenylhydrazine (PHZ) and 5-azacytidine (AZA) according to this procedure [12]. In this manner we wished to validate the dual reporter mice for *in vivo* studies with more direct clinical relevance. The treatment consisted of PHZ intraperitoneal (i.p.) injections the first two days followed by AZA i.p. injections the next five consecutive days. Control WT and transgenic mice were either injected with PHZ followed by PBS instead of AZA (data not shown) or PBS only throughout the seven days of treatment (control mice).

At the end of treatment, blood was analyzed by flow cytometry and bone marrow cells were differentiated *in vitro* in hanging drop (HD) cultures [17] prior to flow cytometry analysis.

**Figure 4:**
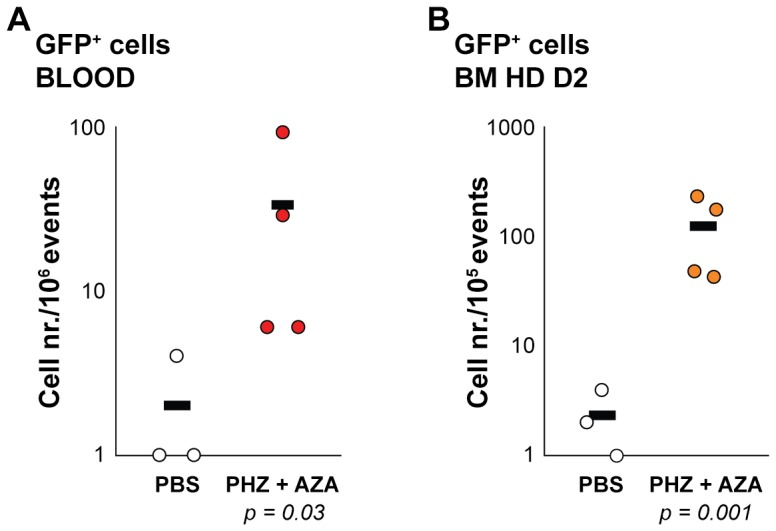
*In vivo* treatment of transgenic mice with 5-Azacytidine. (**A**) Graph representing absolute numbers of GFP^+^ cells/10^6^ events measured gated in blood of γGPA-GFP/βDsRed mice treated (PHZ+AZA, red dots) or mock treated (PBS, white dots). (**B**) Graph representing absolute numbers of GFP^+^ cells/10^5^ events measured after 2 days of bone marrow hanging drop culture (BM HD D2) derived from γGPA-GFP/βDsRed mice treated (PHZ+AZA, orange dots) or mock treated (PBS, white dots). A logarithmic scale is used to better visualize the distribution of values found (each dot represents a mouse). The average per group is depicted as a black line. *P* values were calculated from the Log transformed data with a T-test.

As shown in Figure 4A, treated transgenic mice displayed an increased number of GFP^+^ cells as opposed to mock-treated transgenic mice, indicating that stress erythropoiesis caused by induced hemolytic anemia and followed by AZA administration reactivates the production of human γ-globin in the mouse [12], [23].

The low percentage of F-cells [24] expected in blood during a stress response correlates with the numbers of GFP^+^ cells detected by flow cytometry. Furthermore, we detected GFP^+^ cells co-expressing DsRed (Figure S1A, Table S1).

These results were further verified by culturing bone marrow cells of treated mice in hanging drops (HD) where terminal differentiation occurs more synchronously and globin reporter expression detection is favored, as demonstrated in Figure 3C. At Day 2 of HD culture, the cells were analyzed by flow cytometry. This revealed that the number of GFP^+^ cells is much higher in bone marrow cells derived from treated γGPA-GFP/βDsRed mice when compared to those derived from mock treated mice (Figure 4B). The pattern was similar to that obtained directly from peripheral blood (Figure S1B and Table S2, cells co-expressing GFP and DsRed). Interestingly, compared to the measurements of whole blood, we required 10- to 50-fold fewer cells for flow cytometry analysis of the HD cultures in order to gate sufficient GFP^+^ events for statistical analysis, and the background or auto-fluorescence was reduced. Most importantly, the HD assay can be used in high-throughput assays, requiring minimum amounts of material of both cells and testing agents.

### Transgenic fetal liver cell lines and *in vitro* assays

With the purpose of obtaining stable cell lines for molecular studies, we generated mouse fetal liver cell lines from transgenic embryos (11.5*dpc*–14.5*dpc*) and control (WT) cell lines from littermates after crossing them to homozygosity to the p53 knockout background as described [15], [16].

**Figure 5:**
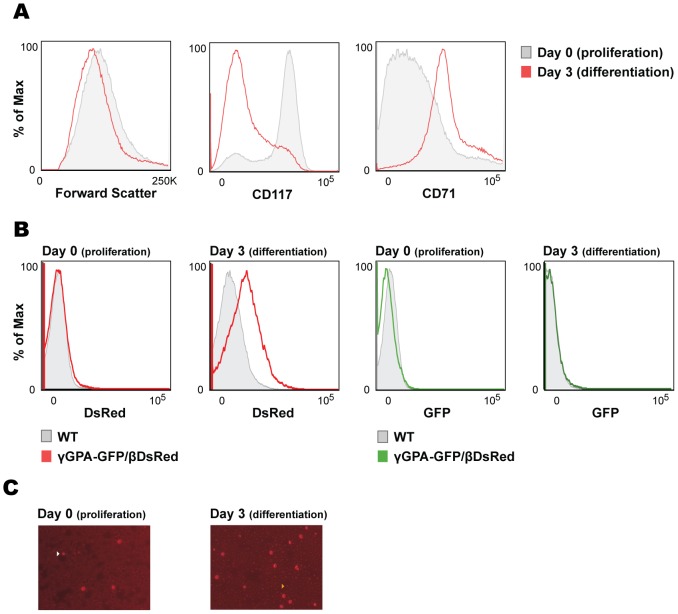
Characterization of the transgenic dual reporter fetal liver cell lines. (**A**) Flow cytometry analysis of transgenic fetal liver cell lines before and after differentiation. Histograms against forward scatter and erythroid surface markers CD117 (cKit) and CD71 (transferrin receptor) are depicted. (**B**) Flow cytometry analysis of transgenic fetal liver cell lines before and after differentiation. Histograms against DsRed and GFP are depicted. (**C**) Representative pictures taken during erythroid differentiation of transgenic fetal liver cell lines. Arrows indicate spontaneously differentiating cells expressing DsRed protein (left) and differentiated cells with much smaller size that are not as bright as the bigger ones, as a consequence of the continuous production of endogenous hemoglobin and subsequent quenching of cytoplasmic DsRed fluorescent signal (right).

The cells can be kept in culture continuously with serum-free media supplemented with erythropoietin (Epo), stem cell factor (SCF) and dexamethasone (Dex). They divide as normal primary mouse fetal liver cells and can be easily driven to differentiation upon replacement of the Epo, SCF and Dex by transferrin and a higher dose of Epo. Most importantly, the differentiation of the cells is homogeneous as reflected by size reduction towards terminal differentiation and production of adult mouse globins (βmaj, data not shown; [15]). Accordingly, cell lines generated from the transgenic mouse lines reduced their size (Figure 5A) and expressed the DsRed protein upon differentiation (Figure 5B and C), which confirms proper protein production of the inserted DsRed cDNA in the β-globin locus.

Flow cytometry analysis of specific erythroid cell membrane markers confirmed the early erythroid progenitor stage of the cells in culture (CD117^+^ CD71^low^). Upon differentiation, cKit (CD117) was downregulated and transferrin receptor (CD71) expression was upregulated (Figure 5A), confirming their capability to undergo normal erythroid differentiation.

We have used the fetal liver cells (primary cells and the established cell lines) from both transgenic lines in several assays, in order to assess their response upon treatment in culture and most importantly their sensitivity to such assays.

We determined the effects of hydroxyurea (HU), AZA or RB7 (short chain fatty acid, SCFA) treatment [10], [12], [25]–[27] on γ-globin reactivation (Figure S2). To our surprise, most of the conditions induced morphological changes in erythroid cells, which resulted in auto-fluorescence of WT treated cultures. This auto-fluorescence interfered with GFP detection (Figure S2A). Therefore we proceeded to analyze GFP transcript induction by qPCR, and normalized the relative fold enrichment by setting respective controls to 1. In agreement with previous work [10], [12], [25]–[27] all treatments induced GFP expression, *i.e.* γ-globin reactivation, and AZA was the most potent activator (Figure S2B).

**Figure 6:**
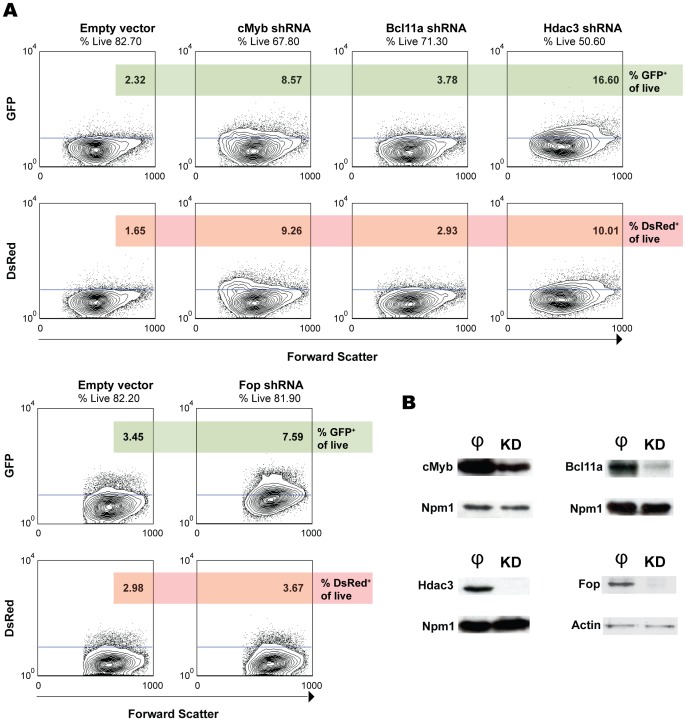
Knockdown assays in transgenic dual reporter fetal liver cell lines. (**A**) Flow cytometry analysis of the knockdown of cMyb, Bcl11a, Hdac3 and Fop in the γGFP/βDsRed cell line. The same vector with a non-specific shRNA sequence was used as a control. Percentages of cells positive for GFP (upper panel) and DsRed (lower panel) are shown. Contour plots show gated live cells. (**B**) Western blots of the knockdown experiments in protein extracts of transduced cells. Equal numbers of cells are loaded on each lane. **φ**, empty vector control extracts; KD, knockdown extracts.

We then tested several shRNA clones for specific downregulation of potential target genes implicated in the regulation of γ-globin gene expression. We have tried several genes, some of which are presented in Figure 6. Knockdown of cMyb [28], Bcl11a [29], Hdac3 [26] and Friend of Prmt1 (Fop) [30] resulted in low, moderate or high induction of GFP expression (Figure 6A). Knockdown efficiencies were confirmed by Western blot analysis (Figure 6B).

cMyb is known to act as an inhibitor of terminal erythroid differentiation [31]. Knockdown of cMyb resulted in GFP (γ-globin) induction (although at low levels) but also DsRed (β-globin) because the cells underwent differentiation as a result of reduced protein levels of cMyb. Conversely, overexpression of cMyb delayed terminal differentiation *in vitro* (data not shown), precluding expression of globins.

Bcl11a knockdown increased GFP expression. However, the GFP expression was not accompanied by DsRed expression to the same extent as observed with cMyb knockdown (GFP/DsRed normalized to controls ratios of Bcl11a and cMyb are 0.9 vs 0.7), consistent with the notion that Bcl11a is a direct target for γ-globin reactivation [28], [29], [32].

Hdac3 knockdown resulted in high GFP levels in every transgenic cell line (Figure 6 and data not shown) and induction of DsRed protein levels to a lower extent (GFP/DsRed normalized to controls ratio 1.2).

Of note, DsRed expression in cMyb and Hdac3 knockdown experiments is confined to different populations of cells when comparing the forward scatter contour plots. In the case of the cMyb knockdown cells, a DsRed-positive population emerges in the low Forward Scatter (FSC) area, a clear indication of cell differentiation. Distinctly, the DsRed positive cells in the Hdac3 knockdown are found in the mid-high FSC area, as occurs with GFP positive cells. This indicates that Hdac3 knockdown results in expression of globins independently of cell differentiation and due to epigenetic changes in the chromatin. This result is in concordance with the co-expression of GFP and DsRed in circulating erythroid cells of PHZ+AZA treated mice (Figure 4 and Figure S1), as AZA also acts modifying the epigenetic signature of chromatin by inhibiting methyltransferases [23], [33] and might induce downregulation of Hdac1 and Hdac3 [34].

Finally, when Friend of Prmt1 (Fop) expression was inhibited we observed an increase in the GFP levels represented by almost all cells (Figure 6A) (GFP/DsRed normalized to controls ratio 1.8). This is in accordance with recently published data [30].

The performed knockdown experiments demonstrate that the cell lines derived from primary cells of transgenic mice respond in agreement with the literature upon manipulation of the expression levels of transcription factors, which are known to influence the erythroid differentiation status in addition to the globin gene expression. The generated cell lines resemble definitive erythropoietic progenitors, i.e. they are at the developmental stage expressing β-globin. Therefore, these cells are a useful tool to study the activation of globin genes and in particular the reactivation of γ-globin (GFP).

## Discussion

In order to understand how globin genes are expressed during each developmental stage several *in vivo* and *in vitro* models have been generated [2], [9]–[13], [25], [26], [35], [36]. The complexity of human globin switching from the fetal γ-globin to the adult β-globin has raised multiple questions that need to be addressed by accurate and flexible models allowing application of high-throughput techniques. Erythroid cell lines such as MEL and K562 have been extensively used and genetically manipulated to provide evidence of globin gene regulation in mouse and human *in vitro* models [10], [11]. The generation of transgenic mice bearing the human β-globin locus allowed dissection of the developmental pattern of globin gene expression and regulation, displaying many similarities but also differences in globin gene regulation between mouse and human [2], [29]. The use of reporter assays to measure the activity of specific promoters under certain conditions that mimic developmental programs of specific cell types is a classical strategy to study transcriptional regulation [37].

Previously, several constructs using fluorescent reporters have been used in the context of a mini-locus construct in mouse (GM979, MEL) [8], [38] and human cell lines (K562) [11], [39] by standard transfection techniques. The main disadvantages of these studies lie in the position effect variegation of the transgene and the integrity of the constructs after the transfection procedure when the whole β-globin locus is used. Another major point is the selective use of promoter regions and hypersensitive sites of the LCR for the generation of reporter constructs that, considering the complex regulation of globin genes, can be suboptimal. A recent example is provided by the Bcl11a protein, which does not appear to bind the γ-globin promoters but has a major effect on human γ-globin expression when knocked down in human proerythroblasts [32] and in Bcl11a knockout mice [29]. Recently, cell lines containing the complete β-globin locus have been generated [10]. However, the copy number of the transgene is not defined in the latter publication and the use of chemical compounds is not tested in WT cells for auto-fluorescence, crucial parameters according to our studies. Furthermore, whilst such erythroleukemic cell lines represent many aspects of erythropoiesis as it occurs *in vivo*, the exogenous transgene is not taken through the epigenetic reprogramming occurring during development of the definitive erythroid system.

This drawback has been overcome by the transgenic mouse lines carrying the modified β-globin locus presented in this study. The mice were analyzed for integrity of the transgenes and for developmental regulation of the reporter genes, which contrary to what has been described by Chan and coworkers, do not replace the endogenous globin genes. To minimize quenching of fluorescence due to the highly absorbent hemoglobin environment of the definitive red cell we used the fusion γGPA-GFP/βDsRed construct. Expression of GPA-GFP on the erythroid cell outer membrane provided clues about the limits and sensitivity of the dual reporter system *in vivo* and *in vitro*. Such a strategy has been used by others, who chose instead the nucleus to locate the GFP fluorescent protein by fusing it to H2B histone [36]. This way, Isern and coworkers [36] marked primitive erythroid cells since they expressed it under the regulatory elements of the human *HBE* promoter. In our model system, and as shown by others, we could assign γ-globin expression to primitive erythroid cells based on flow cytometry analysis of 11.5*dpc*–14.5*dpc* fetal blood [29].

The problem of fluorescence quenching in the red cells has been noticed previously [21], [35], [37], [40] and is difficult to overcome especially when moderate levels of expression are achieved, *i.e.* with low copy number of the transgene and even more important, in the case of γ-globin reactivation. Our study emphasizes particularly on both issues by comparing the two mouse lines (γGFP/βDsRed versus γGPA-GFP/βDsRed)keeping the copies of the transgene at minimum, one or two copies.

We noticed an additional problem when agents such as 5-azacytidine (AZA), hydroxyurea (HU) or RB7 were used and also with other Hdac inhibitors (data not shown). These agents changed the morphology of the cells dramatically and exerted significant levels of auto-fluorescence in WT cells. This is a limitation that has to be taken in consideration when such experiments are performed. Therefore, despite the GPA-GFP protein placed in the plasma membrane when expressed *in vivo* in embryonic blood improved the detection of fluorescence signals, caution should be taken when using agents that can induce auto-fluorescence in the cells under study. Therefore, a WT cell line should be always taken along as a negative control.

Despite this limitation, the potential use of this system is broad and it has the unique advantage that it allows sorting of GFP-positive (γ-globin) cells for further analysis. First, we show by culturing fetal liver and bone marrow in hanging drops (HD) [17], which go through terminal differentiation in a more synchronous manner that globin reporter expression detection is favored as compared to primary material, suggesting that the HD cultures can be used in high-throughput assays, requiring minimum amounts of material of both cells and testing agents and overcome the noise caused by fully hemoglobinized cells in primary tissues. Screens of libraries of shRNA clones or synthetic antibodies are unbiased approaches that can be applied to the dual reporter β-globin locus cell lines. Thus, these lines provide a tool for identification of changes at the molecular level in a population of cells or at single cell level using high-throughput assays. When potential candidate compounds or drugs proven to reactivate γ-globin *in vitro* are selected, translational studies could be performed with the mouse lines. As an example, we demonstrated detection of GFP^+^ cells upon stress erythropoiesis induction followed by AZA treatment. Of note, we observed that the GFP^+^ cells co-expressed DsRed, an observation that would be missed when analyzing RNA isolated from the pool of circulating reticulocytes. In concordance with this result, we also found co-expression of DsRed and GFP *in vitro* when knocking down Hdac3, which is not surprising since AZA also acts modifying the epigenetic signature of chromatin by inhibiting methyltransferases [23], [33] and might induce downregulation of Hdac1 and Hdac3 [34].

In our *in vitro* cultures of transgenic fetal liver cells in the presence of AZA or HU, we could detect by qPCR upregulation of DsRed transcript, although not at the same extent as GFP transcript (data not shown and Figure S2B). Unfortunately, due to the auto-fluorescence induced in the cells, it was not possible to assess by flow cytometry whether the cells expressing GFP do co-express DsRed. This limitation could be partly overcome by choosing new developed fluorescent proteins (e.g. mNeptune) with emission wavelengths in the far red, highly stable and photobleaching resistant. This would reduce auto-fluorescence and quenching due to hemoglobin in these spectra or at least minimize it at levels that do not compromise the results in high-throughput assays [22]. However, these “improved” fluorescent proteins have to be tested *in vivo* for their functionality. Our decision on choosing the DsRed2 protein for its emission wavelength at 582 nm was borderline as we observed when using chemical compounds with unexpected absorbing characteristics or when erythroid cells matured and enucleated. Structural studies for the development of brighter proteins in combination with physical separation of interacting molecules that quench fluorescent signals, as we intend here by fusing the GFP protein with the membrane GPA, establish the basis for the future generation of reporter mouse models.

It is important to mention that both *in vitro* and *in vivo* assays with our reporter system have explored for the first time the possibility of imaging the expression of globin genes upon minimum manipulation of their genomic context. This is crucial in order to avoid artifacts and overrating results. Here, we demonstrate that the efforts for the generation of an optimal reporter system have to develop further in order to reach maximum sensitivity. In the meantime, our reporter mice and cell lines provide the necessary tools for performing reporter assays regarding globin gene expression as we show by a series of different experiments.

### Concluding remarks

We have generated a dual reporter human β-globin locus mouse model in order to trace the expression of the two β-like globin genes involved in the fetal to adult switch, i.e. γ- and β-globin genes, while within their intact genomic context. We have generated from these mouse lines immortalized erythroid transgenic fetal liver cell lines, which allow *in vitro* studies and it is possible to monitor globin gene expression at the proerythroblast stage or during differentiation. Such a reporter cell line represents a physiological analogue of an erythroid progenitor in terms of proper transcriptional regulation, indispensable for developmental studies of globin expression and unbiased high-throughput screens. Such experiments are limited when agents used in the culture alter cell morphology and/or induce auto-fluorescence, therefore we propose to conduct them with WT cells as negative control. More importantly, when identifying potential candidates of γ-globin reactivation, the mouse models generated can be used for *in vivo* experiments, where GFP^+^ cells can be traced directly in an unbiased manner from circulating erythroid cells or from bone marrow hanging drop cultures, highlighting its potential applied and clinical relevance. A compromise has to be taken when generating reporter mouse models for transcription regulation studies regarding intact regulatory sequences and low copy number of the transgene as well as expression levels and structural properties of the fluorescent protein.

## Supporting Information

Figure S1 ***In vivo*** **treatment of transgenic mice with 5-Azacytidine.** (**A**) Flow cytometry analysis of PHZ+AZA administration in transgenic mice (γGPA-GFP/βDsRed). The upper two contour plots show background levels of fluorescence in peripheral blood upon PBS administration. The lower panel shows the response of WT and γGPA-GFP/βDsRed mice upon PHZ and AZA administration. (**B**) Bone marrow hanging drop culture of the treated mice, presented in the same order as above (A).
(TIF)

Figure S2 **reatment of transgenic dual reporter fetal liver cell lines with chemical compounds.** (**A**) Flow cytometry analysis of WT and transgenic fetal liver cell lines treated or untreated with HU, AZA or RB7. Histogram overlays against GFP are depicted. (**B**) qPCR analysis of GFP expression in WT and transgenic fetal liver cell lines treated or untreated with HU, AZA or RB7. RFE is relative fold enrichment. Average and standard deviation from 3 independent experiments are depicted after normalization of the untreated or DMSO controls. T-test was performed to calculate the *p* values.
(TIF)

Table S1 **Absolute cell numbers from blood of PHZ+AZA/PBS treated mice.** Blood of treated (PHZ + AZA) or mock treated (PBS) mice was analyzed by flow cytometry and cells positive for DsRed, GFP and DsRed+GFP were counted.
(TIF)

Table S2 **Absolute cell numbers from bone marrow hanging drops of PHZ+AZA/PBS treated mice.** Bone marrow cells from treated (PHZ + AZA) or mock treated (PBS) mice were differentiated in hanging drops (HD) for two days and analyzed by flow cytometry. Cells positive for DsRed, GFP and DsRed+GFP were counted.
(TIF)
