# Supplementary figures and images for: A Dual Reporter Mouse Model of the Human β-Globin Locus: Applications and Limitations
Source: PLoS One. 2012 Dec 14;7(12):e51272. doi: 10.1371/journal.pone.0051272 (PMC3522686; doi:10.1371/journal.pone.0051272)

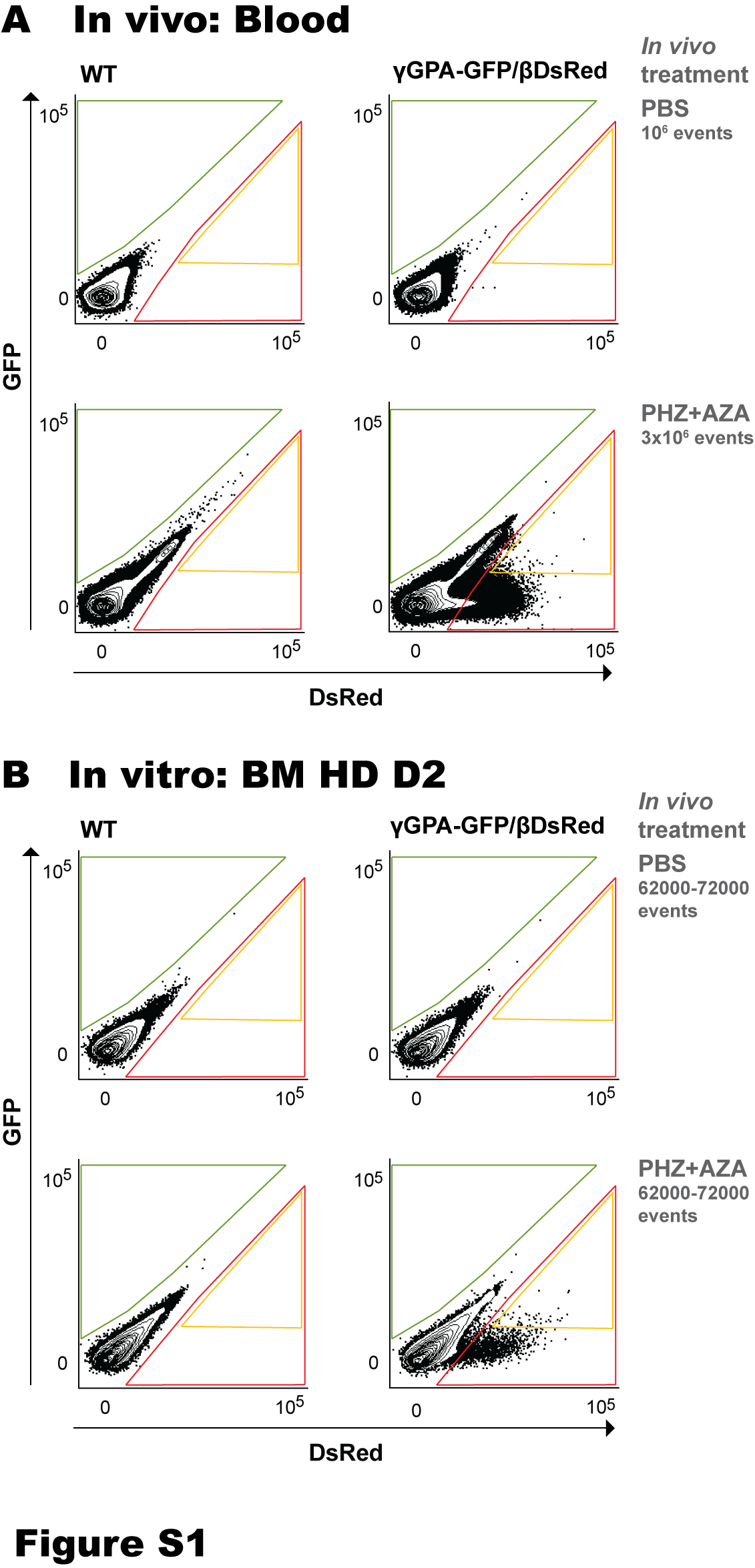

Supplement: Figure S1 — In vivo treatment of transgenic mice with 5-Azacytidine. (A) Flow cytometry analysis of PHZ+AZA administration in transgenic mice (γGPA-GFP/βDsRed). The upper two contour plots show background levels of fluorescence in peripheral blood upon PBS administration. The lower panel shows the response of WT and γGPA-GFP/βDsRed mice upon PHZ and AZA administration. (B) Bone marrow hanging drop culture of the treated mice, presented in the same order as above (A). (TIF) [file pone.0051272.s001.tif]

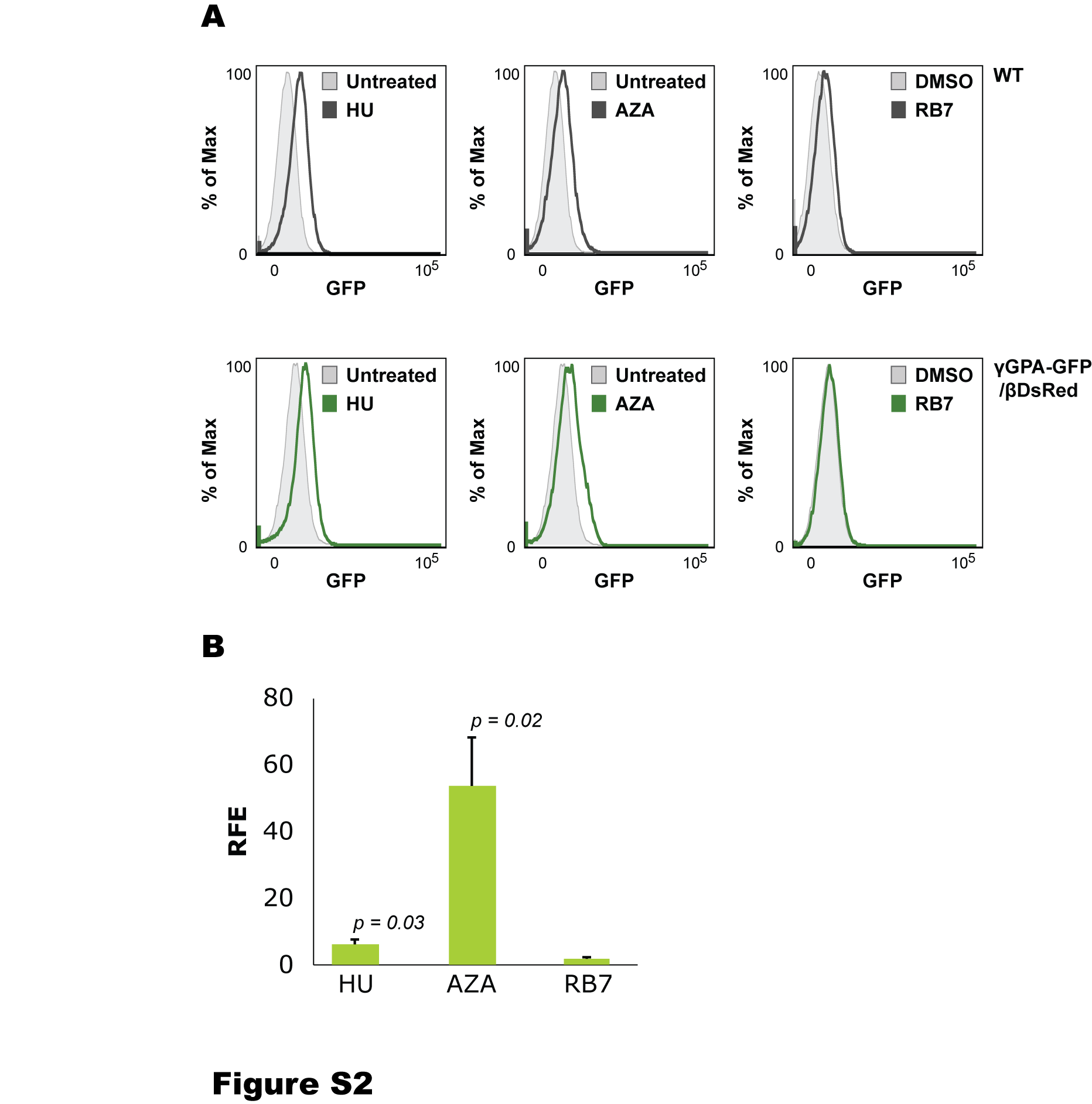

Supplement: Figure S2 — reatment of transgenic dual reporter fetal liver cell lines with chemical compounds. (A) Flow cytometry analysis of WT and transgenic fetal liver cell lines treated or untreated with HU, AZA or RB7. Histogram overlays against GFP are depicted. (B) qPCR analysis of GFP expression in WT and transgenic fetal liver cell lines treated or untreated with HU, AZA or RB7. RFE is relative fold enrichment. Average and standard deviation from 3 independent experiments are depicted after normalization of the untreated or DMSO controls. T-test was performed to calculate the p values. (TIF) [file pone.0051272.s002.tif]

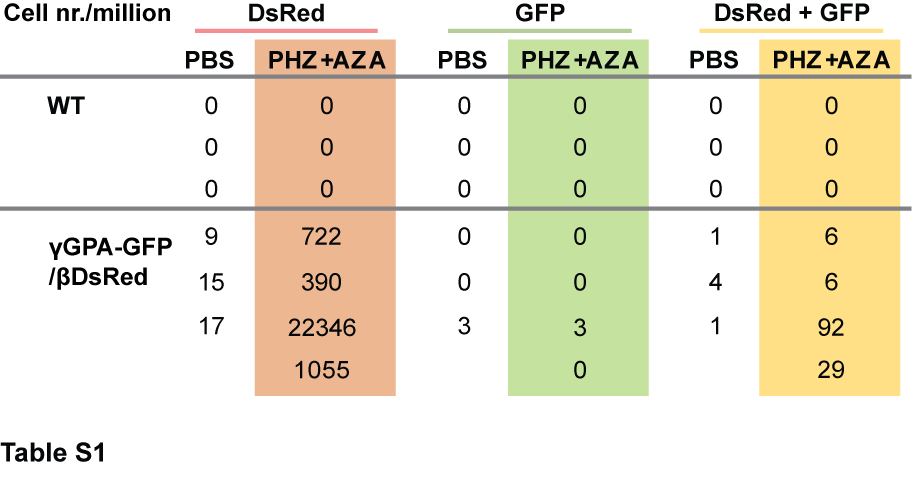

Supplement: Table S1 — Absolute cell numbers from blood of PHZ+AZA/PBS treated mice. Blood of treated (PHZ + AZA) or mock treated (PBS) mice was analyzed by flow cytometry and cells positive for DsRed, GFP and DsRed+GFP were counted. (TIF) [file pone.0051272.s003.tif]

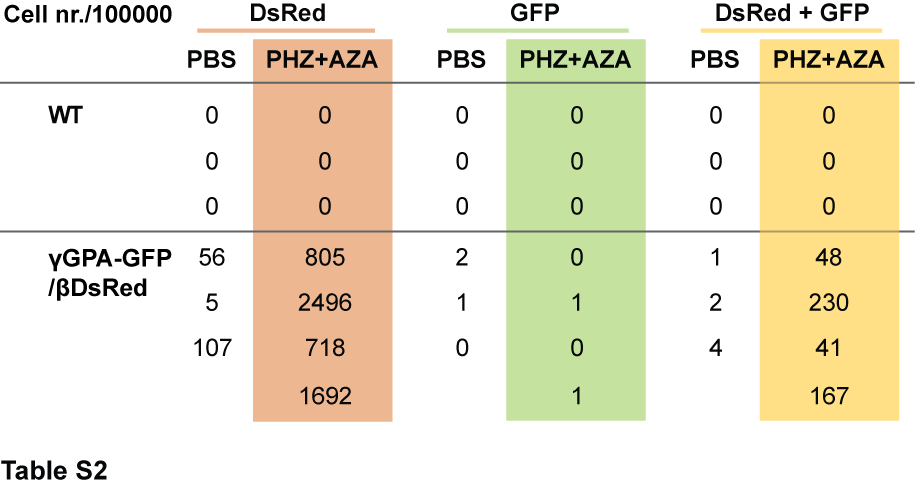

Supplement: Table S2 — Absolute cell numbers from bone marrow hanging drops of PHZ+AZA/PBS treated mice. Bone marrow cells from treated (PHZ + AZA) or mock treated (PBS) mice were differentiated in hanging drops (HD) for two days and analyzed by flow cytometry. Cells positive for DsRed, GFP and DsRed+GFP were counted. (TIF) [file pone.0051272.s004.tif]
